# (3aR*,5R*)-5-(4-Chloro­phen­yl)-1,2,3,3a-tetra­hydro­benzo[*e*]pyrrolo­[2,1-*b*][1,3]ox­azepin-10(5*H*)-one

**DOI:** 10.1107/S1600536811020265

**Published:** 2011-06-11

**Authors:** Yun-Zhou Jin, Rong-Hua Zhang, Da-Xu Fu, Yao-Kang Lv

**Affiliations:** a Chemistry Department, Tongji University, Shanghai 200092, People’s Republic of China

## Abstract

The title compound, C_18_H_16_ClNO_2_, is the main product of a photoreaction. The two benzene rings make a dihedral angle of 86.40 (2)° with each other. The 1,3-oxazepine C atom to which the 4-chloro­phenyl group is attached and the C atom of the 4-chloro­phenyl group attached to the 1,3-oxazepine ring are chiral C atoms, but the crystal is a racemate in which the enanti­omers are linked by a pair of weak inter­molecular C—H⋯O hydrogen bond, forming an inversion dimer.

## Related literature

For general background to asymmetric photochemical reactions, see: Gratzel (2001 ▶); Korzeniewski & Zoladz (2001 ▶); Aubert *et al.* (2000 ▶). For photo-induced cyclizations, see Griesbeck *et al.* (2002 ▶); Henz *et al.* (1995 ▶). For related structures, see: Griesbeck *et al.* (1997 ▶, 1999 ▶); Basarić *et al.* (2008 ▶).
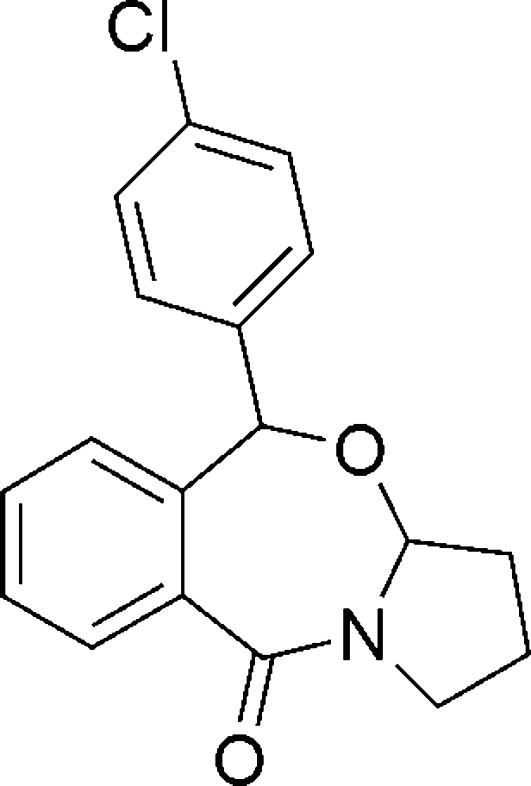


## Experimental

### 

#### Crystal data

C_18_H_16_ClNO_2_
*M* *_r_* = 313.77
Monoclinic, 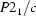

*a* = 8.1764 (6) Å
*b* = 16.9030 (11) Å
*c* = 11.1564 (8) Å
β = 98.224 (6)°
*V* = 1526.02 (19) Å^3^
*Z* = 4
Mo *K*α radiation
μ = 0.26 mm^−1^
*T* = 296 K
0.22 × 0.18 × 0.15 mm

#### Data collection

Bruker APEXII area-detector diffractometer
Absorption correction: multi-scan (*SADABS*; Sheldrick, 1996 ▶) *T*_min_ = 0.676, *T*_max_ = 1.000
12942 measured reflections
3478 independent reflections
2393 reflections with *I* > 2σ(*I*)
*R*_int_ = 0.039

#### Refinement

*R*[*F* ^2^ > 2σ(*F* ^2^)] = 0.047
*wR*(*F* ^2^) = 0.181
*S* = 1.04
3478 reflections
199 parameters
H-atom parameters constrained
Δρ_max_ = 0.31 e Å^−3^
Δρ_min_ = −0.37 e Å^−3^

### 

Data collection: *APEX2* (Bruker, 2006 ▶); cell refinement: *SAINT* (Bruker, 2006 ▶); data reduction: *SAINT*; program(s) used to solve structure: *SHELXS97* (Sheldrick, 2008 ▶); program(s) used to refine structure: *SHELXL97* (Sheldrick, 2008 ▶); molecular graphics: *DIAMOND* (Brandenburg & Putz, 2004 ▶); software used to prepare material for publication: *SHELXTL* (Sheldrick, 2008 ▶).

## Supplementary Material

Crystal structure: contains datablock(s) I, global. DOI: 10.1107/S1600536811020265/ff2014sup1.cif

Structure factors: contains datablock(s) I. DOI: 10.1107/S1600536811020265/ff2014Isup2.hkl

Supplementary material file. DOI: 10.1107/S1600536811020265/ff2014Isup3.cml

Additional supplementary materials:  crystallographic information; 3D view; checkCIF report

## Figures and Tables

**Table 1:** Hydrogen-bond geometry (Å, °)

*D*—H⋯*A*	*D*—H	H⋯*A*	*D*⋯*A*	*D*—H⋯*A*
C12—H12*A*⋯O1^i^	0.98	2.27	3.206 (3)	159

Symmetry code: (i) 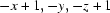.

**Fig. 1.**
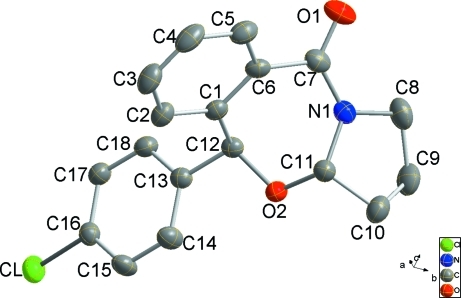
The molecular structure of the title compound with displacement ellipsoids drawn at the 50% probability level. H atoms are omitted for clarity.

**Fig. 2.**
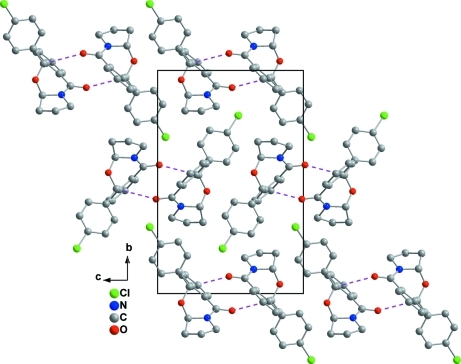
Partial packing diagram showing the C—H···O interaction in the racemate.

**Fig. 3.**
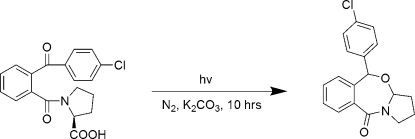
Reaction scheme of the preparation of the title compound.

## supplementary crystallographic information

### Comment

In modern organic chemistry preparative organic photochemistry is an important tool to synthesize complex compounds in one step.(Gratzel, 2001; Korzeniewski & Zoladz, 2001; Aubert *et al.*, 2000). Benzophenone acylamide derivatives can form the seven-membered ring through the intramolecular photoinduced decarboxylation and cyclization (Griesbeck *et al.*, 2002; Henz *et al.*,1995). We report herein the crystal structure and synthesis of the title compound.

The structure of the title compound is shown in Fig.1. The dihedral angle between the two benzene rings is 86.40 (2). Atoms C11 and C12 of the title compound are chiral, but the compound crystallizes as a racemate, in which *R* and *S* pairs are linked by pairs of weak intermolecular C—H···O hydrogen bonds (Fig. 2).

### Experimental

The title compound was the main product of the photoreaction of (*S*)-1-(2-(4-chlorobenzoyl) benzoyl)pyrrolidine-2-carboxylic acid under N_2_ for 10 h (Fig.3). The compound was purified by flash column chromatography (silica gel column, petroleum ether/ethyl acetate=4/1). Colourless crystals for the X-ray crystallographic studies were gained from slow evaporation of a dichloromethane solution.

### Refinement

The structure was solved by direct methods and expanded with difference Fourier techniques. All non-hydrogen atoms were refined anisotropically by the full matrix least-squares on the *F*^2^. The hydrogen atoms attached to carbon atoms were located by geometrical calculation using a riding model [*U*_iso_(H) = 1.2*U*_eq_(C)].

### Crystal data

**Table:**

C_18_H_16_ClNO_2_	*F*(000) = 656
*M**_r_* = 313.77	*D*_x_ = 1.366 Mg m^−^^3^
Monoclinic, *P*2_1_/*c*	Mo *K*α radiation, λ = 0.71073 Å
Hall symbol: -P 2ybc	Cell parameters from 3950 reflections
*a* = 8.1764 (6) Å	θ = 2.2–27.5°
*b* = 16.9030 (11) Å	µ = 0.26 mm^−^^1^
*c* = 11.1564 (8) Å	*T* = 296 K
β = 98.224 (6)°	Prism, colourless
*V* = 1526.02 (19) Å^3^	0.22 × 0.18 × 0.15 mm
*Z* = 4	

### Data collection

**Table:**

Bruker APEXII area-detector diffractometer	3478 independent reflections
Radiation source: fine-focus sealed tube	2393 reflections with *I* > 2σ(*I*)
graphite	*R*_int_ = 0.039
ω scans	θ_max_ = 27.5°, θ_min_ = 2.2°
Absorption correction: multi-scan (*SADABS*; Sheldrick, 1996)	*h* = −10→10
*T*_min_ = 0.676, *T*_max_ = 1.000	*k* = −21→19
12942 measured reflections	*l* = −14→14

### Refinement

**Table:**

Refinement on *F*^2^	Primary atom site location: structure-invariant direct methods
Least-squares matrix: full	Secondary atom site location: difference Fourier map
*R*[*F*^2^ > 2σ(*F*^2^)] = 0.047	Hydrogen site location: inferred from neighbouring sites
*wR*(*F*^2^) = 0.181	H-atom parameters constrained
*S* = 1.04	*w* = 1/[σ^2^(*F*_o_^2^) + (0.092*P*)^2^] where *P* = (*F*_o_^2^ + 2*F*_c_^2^)/3
3478 reflections	(Δ/σ)_max_ < 0.001
199 parameters	Δρ_max_ = 0.31 e Å^−^^3^
0 restraints	Δρ_min_ = −0.37 e Å^−^^3^

### Special details

**Table:**

Geometry. All e.s.d.'s (except the e.s.d. in the dihedral angle between two l.s. planes) are estimated using the full covariance matrix. The cell e.s.d.'s are taken into account individually in the estimation of e.s.d.'s in distances, angles and torsion angles; correlations between e.s.d.'s in cell parameters are only used when they are defined by crystal symmetry. An approximate (isotropic) treatment of cell e.s.d.'s is used for estimating e.s.d.'s involving l.s. planes.
Refinement. Refinement of *F*^2^ against ALL reflections. The weighted *R*-factor *wR* and goodness of fit *S* are based on *F*^2^, conventional *R*-factors *R* are based on *F*, with *F* set to zero for negative *F*^2^. The threshold expression of *F*^2^ > σ(*F*^2^) is used only for calculating *R*-factors(gt) *etc*. and is not relevant to the choice of reflections for refinement. *R*-factors based on *F*^2^ are statistically about twice as large as those based on *F*, and *R*- factors based on ALL data will be even larger.

### Fractional atomic coordinates and isotropic or equivalent isotropic displacement parameters (Å^2^)

**Table:**

	*x*	*y*	*z*	*U*_iso_*/*U*_eq_	
Cl1	0.03682 (9)	−0.29821 (4)	−0.05399 (7)	0.0622 (3)	
N1	0.4690 (3)	0.10862 (13)	0.34094 (18)	0.0480 (5)	
C1	0.5968 (3)	−0.03368 (14)	0.2353 (2)	0.0413 (6)	
O1	0.6417 (2)	0.07672 (14)	0.51050 (16)	0.0688 (6)	
O2	0.3452 (2)	0.04181 (10)	0.16210 (15)	0.0489 (5)	
C2	0.6842 (3)	−0.08243 (15)	0.1681 (2)	0.0499 (6)	
H2A	0.6277	−0.1146	0.1084	0.060*	
C3	0.8560 (3)	−0.08382 (18)	0.1887 (3)	0.0651 (8)	
H3A	0.9132	−0.1165	0.1420	0.078*	
C4	0.9415 (3)	−0.03783 (18)	0.2764 (3)	0.0683 (9)	
H4A	1.0564	−0.0392	0.2897	0.082*	
C5	0.8567 (3)	0.01065 (17)	0.3453 (3)	0.0574 (7)	
H5A	0.9148	0.0415	0.4060	0.069*	
C6	0.6849 (3)	0.01392 (15)	0.3250 (2)	0.0445 (6)	
C7	0.5983 (3)	0.06870 (16)	0.4016 (2)	0.0501 (6)	
C8	0.3617 (4)	0.16033 (19)	0.4019 (3)	0.0692 (9)	
H8A	0.4206	0.2071	0.4349	0.083*	
H8B	0.3182	0.1325	0.4665	0.083*	
C9	0.2247 (4)	0.1818 (2)	0.2998 (3)	0.0719 (9)	
H9A	0.1359	0.1433	0.2931	0.086*	
H9B	0.1801	0.2338	0.3121	0.086*	
C10	0.3120 (4)	0.18035 (17)	0.1886 (3)	0.0631 (8)	
H10A	0.2336	0.1728	0.1157	0.076*	
H10B	0.3719	0.2293	0.1814	0.076*	
C11	0.4297 (3)	0.11097 (15)	0.2095 (2)	0.0461 (6)	
H11A	0.5292	0.1201	0.1718	0.055*	
C12	0.4073 (3)	−0.03092 (14)	0.2181 (2)	0.0413 (5)	
H12A	0.3737	−0.0326	0.2989	0.050*	
C13	0.3216 (3)	−0.09805 (14)	0.1466 (2)	0.0415 (5)	
C14	0.2537 (3)	−0.08971 (16)	0.0260 (2)	0.0541 (7)	
H14A	0.2660	−0.0423	−0.0140	0.065*	
C15	0.1679 (3)	−0.15155 (16)	−0.0348 (2)	0.0545 (7)	
H15A	0.1214	−0.1451	−0.1153	0.065*	
C16	0.1508 (3)	−0.22169 (14)	0.0218 (2)	0.0456 (6)	
C17	0.2183 (3)	−0.23170 (16)	0.1421 (2)	0.0500 (6)	
H17A	0.2075	−0.2797	0.1809	0.060*	
C18	0.3018 (3)	−0.16948 (16)	0.2039 (2)	0.0488 (6)	
H18A	0.3454	−0.1756	0.2850	0.059*	

### Atomic displacement parameters (Å^2^)

**Table:**

	*U*^11^	*U*^22^	*U*^33^	*U*^12^	*U*^13^	*U*^23^
Cl1	0.0607 (4)	0.0489 (4)	0.0745 (5)	−0.0033 (3)	0.0014 (4)	−0.0117 (3)
N1	0.0496 (12)	0.0520 (12)	0.0433 (11)	−0.0005 (10)	0.0095 (9)	−0.0059 (9)
C1	0.0387 (12)	0.0429 (13)	0.0426 (13)	0.0014 (10)	0.0065 (10)	0.0107 (10)
O1	0.0684 (13)	0.0907 (16)	0.0452 (11)	−0.0246 (11)	0.0010 (9)	−0.0051 (10)
O2	0.0487 (10)	0.0453 (10)	0.0494 (10)	0.0068 (8)	−0.0042 (8)	−0.0017 (8)
C2	0.0471 (13)	0.0444 (13)	0.0594 (16)	0.0057 (11)	0.0119 (12)	0.0081 (11)
C3	0.0515 (15)	0.0518 (16)	0.096 (2)	0.0119 (13)	0.0243 (16)	0.0092 (16)
C4	0.0383 (13)	0.0613 (18)	0.104 (3)	0.0062 (13)	0.0070 (15)	0.0223 (17)
C5	0.0412 (13)	0.0593 (17)	0.0688 (18)	−0.0024 (12)	−0.0019 (13)	0.0132 (14)
C6	0.0417 (12)	0.0508 (14)	0.0398 (13)	−0.0027 (11)	0.0012 (10)	0.0109 (10)
C7	0.0473 (13)	0.0552 (16)	0.0476 (14)	−0.0167 (12)	0.0064 (12)	−0.0008 (12)
C8	0.0699 (19)	0.068 (2)	0.074 (2)	−0.0016 (16)	0.0257 (16)	−0.0235 (16)
C9	0.0612 (17)	0.0620 (19)	0.095 (2)	0.0130 (15)	0.0202 (17)	−0.0128 (17)
C10	0.0603 (17)	0.0490 (15)	0.079 (2)	0.0093 (13)	0.0079 (15)	0.0013 (14)
C11	0.0473 (13)	0.0464 (14)	0.0444 (14)	0.0010 (11)	0.0057 (11)	−0.0002 (11)
C12	0.0390 (11)	0.0472 (13)	0.0383 (12)	0.0004 (10)	0.0070 (10)	0.0011 (10)
C13	0.0373 (11)	0.0466 (13)	0.0408 (12)	0.0008 (10)	0.0066 (10)	0.0007 (10)
C14	0.0646 (16)	0.0526 (15)	0.0444 (14)	−0.0120 (13)	0.0053 (13)	0.0066 (11)
C15	0.0674 (17)	0.0589 (17)	0.0353 (13)	−0.0085 (13)	0.0003 (12)	−0.0006 (11)
C16	0.0412 (12)	0.0443 (13)	0.0514 (14)	0.0013 (10)	0.0066 (11)	−0.0060 (11)
C17	0.0543 (14)	0.0441 (13)	0.0507 (14)	0.0039 (12)	0.0042 (12)	0.0053 (11)
C18	0.0464 (13)	0.0511 (14)	0.0471 (14)	0.0048 (11)	0.0000 (11)	0.0055 (11)

### Geometric parameters (Å, °)

**Table:**

Cl1—C16	1.741 (2)	C8—H8B	0.9700
N1—C7	1.351 (3)	C9—C10	1.517 (4)
N1—C11	1.456 (3)	C9—H9A	0.9700
N1—C8	1.472 (3)	C9—H9B	0.9700
C1—C2	1.381 (4)	C10—C11	1.514 (4)
C1—C6	1.400 (3)	C10—H10A	0.9700
C1—C12	1.534 (3)	C10—H10B	0.9700
O1—C7	1.223 (3)	C11—H11A	0.9800
O2—C11	1.421 (3)	C12—C13	1.502 (3)
O2—C12	1.438 (3)	C12—H12A	0.9800
C2—C3	1.390 (3)	C13—C18	1.386 (3)
C2—H2A	0.9300	C13—C14	1.387 (3)
C3—C4	1.362 (4)	C14—C15	1.382 (3)
C3—H3A	0.9300	C14—H14A	0.9300
C4—C5	1.377 (4)	C15—C16	1.360 (4)
C4—H4A	0.9300	C15—H15A	0.9300
C5—C6	1.392 (3)	C16—C17	1.386 (3)
C5—H5A	0.9300	C17—C18	1.383 (4)
C6—C7	1.505 (4)	C17—H17A	0.9300
C8—C9	1.523 (4)	C18—H18A	0.9300
C8—H8A	0.9700		
			
C7—N1—C11	124.2 (2)	C11—C10—C9	104.4 (2)
C7—N1—C8	122.7 (2)	C11—C10—H10A	110.9
C11—N1—C8	112.9 (2)	C9—C10—H10A	110.9
C2—C1—C6	118.5 (2)	C11—C10—H10B	110.9
C2—C1—C12	122.8 (2)	C9—C10—H10B	110.9
C6—C1—C12	118.6 (2)	H10A—C10—H10B	108.9
C11—O2—C12	114.74 (15)	O2—C11—N1	112.2 (2)
C1—C2—C3	120.6 (3)	O2—C11—C10	108.37 (19)
C1—C2—H2A	119.7	N1—C11—C10	102.7 (2)
C3—C2—H2A	119.7	O2—C11—H11A	111.1
C4—C3—C2	120.8 (3)	N1—C11—H11A	111.1
C4—C3—H3A	119.6	C10—C11—H11A	111.1
C2—C3—H3A	119.6	O2—C12—C13	107.83 (17)
C3—C4—C5	119.5 (3)	O2—C12—C1	111.60 (19)
C3—C4—H4A	120.2	C13—C12—C1	115.5 (2)
C5—C4—H4A	120.2	O2—C12—H12A	107.2
C4—C5—C6	120.7 (3)	C13—C12—H12A	107.2
C4—C5—H5A	119.7	C1—C12—H12A	107.2
C6—C5—H5A	119.7	C18—C13—C14	118.6 (2)
C5—C6—C1	119.9 (3)	C18—C13—C12	119.3 (2)
C5—C6—C7	118.6 (2)	C14—C13—C12	122.0 (2)
C1—C6—C7	121.6 (2)	C15—C14—C13	120.3 (2)
O1—C7—N1	122.5 (3)	C15—C14—H14A	119.9
O1—C7—C6	122.6 (2)	C13—C14—H14A	119.9
N1—C7—C6	114.9 (2)	C16—C15—C14	120.7 (2)
N1—C8—C9	102.7 (2)	C16—C15—H15A	119.7
N1—C8—H8A	111.2	C14—C15—H15A	119.7
C9—C8—H8A	111.2	C15—C16—C17	120.2 (2)
N1—C8—H8B	111.2	C15—C16—Cl1	120.34 (19)
C9—C8—H8B	111.2	C17—C16—Cl1	119.4 (2)
H8A—C8—H8B	109.1	C18—C17—C16	119.3 (2)
C10—C9—C8	103.0 (2)	C18—C17—H17A	120.4
C10—C9—H9A	111.2	C16—C17—H17A	120.4
C8—C9—H9A	111.2	C17—C18—C13	121.0 (2)
C10—C9—H9B	111.2	C17—C18—H18A	119.5
C8—C9—H9B	111.2	C13—C18—H18A	119.5
H9A—C9—H9B	109.1		

### Hydrogen-bond geometry (Å, °)

**Table:**

*D*—H···*A*	*D*—H	H···*A*	*D*···*A*	*D*—H···*A*
C12—H12A···O1^i^	0.98	2.27	3.206 (3)	159

Symmetry codes: (i) −*x*+1, −*y*, −*z*+1.

## References

[bb1] Aubert, C., Vos, M. H., Mathias, P., Eker, A. M. & Brettle, K. (2000). *Nature (London)*, **407**, 926. 10.1038/35014644 10850720

[bb2] Basarić, N., Horvat, M., Mlinarić-Majerski, K., Zimmermann, E., Neudörfl, J. & Griesbeck, A. G. (2008). *Org. Lett.* **10**, 3965–3968. 10.1021/ol801362x 18715013

[bb3] Brandenburg, K. & Putz, H. (2004). *DIAMOND* Crystal Impact GbR, Bonn, Germany.

[bb4] Bruker (2006). *APEX2* and *SAINT* Bruker AXS Inc., Madison, Wisconsin, USA.

[bb5] Gratzel, M. (2001). *Pure Appl. Chem.* **73**, 459–467.

[bb6] Griesbeck, A. G., Heinrich, T., Oelgemöller, M., Molis, A. & Heidtann, A. (2002). *Helv. Chim. Acta*, **85**, 4561–4577.

[bb7] Griesbeck, A. G., Henz, A., Kramer, W., Lex, J., Nerowshi, F. & Oelgemöller, M. (1997). *Helv. Chim. Acta*, **80**, 912–933.

[bb8] Griesbeck, A. G., Nerowski, F. & Lex, J. (1999). *J. Org. Chem.* **64**, 5213–5217. 10.1021/jo990390b 34237854

[bb9] Henz, A., Griesbeck, A. G. & Peters, K. (1995). *Angew. Chem. Int. Ed.* **34**, 474–491.

[bb10] Korzeniewski, B. & Zoladz, J. A. (2001). *Biophys. Chem.* **92**, 7–34. 10.1016/s0301-4622(01)00184-3 11527576

[bb11] Sheldrick, G. M. (1996). *SADABS* University of Göttingen, Germany.

[bb12] Sheldrick, G. M. (2008). *Acta Cryst.* A**64**, 112–122. 10.1107/S0108767307043930 18156677

